# Responding to the need of postgraduate education for Planetary Health: Development of an online Master's Degree

**DOI:** 10.3389/fpubh.2022.969065

**Published:** 2022-10-26

**Authors:** Cristina O'Callaghan-Gordo, Ariadna Moreno, Marina Bosque-Prous, Enrique Castro-Sanchez, Payam Dadvand, Carlos A. Faerron Guzmán, Ana García-Juanatey, Mireia Gascon, Oriol Grau, Jacint Jordana, Rachel Lowe, Hug March, F. Xavier Medina, Lela Mélon, Grettel Navas, Andrea Núñez Casal, Isabel Ruiz-Mallén, Nacho Sánchez-Valdivia, Cathryn Tonne, Margarita Triguero-Mas, Christos Zografos, Josep M. Antó

**Affiliations:** ^1^Faculty of Health Sciences, Universitat Oberta de Catalunya, Barcelona, Spain; ^2^ISGlobal, Barcelona, Spain; ^3^Universitat Pompeu Fabra (UPF), Barcelona, Spain; ^4^CIBER Epidemiología y Salud Pública (CIBERESP), Madrid, Spain; ^5^College of Nursing, Midwifery and Healthcare, University of West London, Brentford, United Kingdom; ^6^Health Protection Research Unit in Healthcare Associated Infection and Antimicrobial Resistance, Imperial College London, London, United Kingdom; ^7^Graduate School, University of Maryland, Baltimore, MD, United States; ^8^Planetary Health Alliance, Harvard T.H. Chan School of Public Health, Boston, MA, United States; ^9^CEI International Affairs, Barcelona, Spain; ^10^Plants and Ecosystems, University of Antwerpen, Antwerpen, Belgium; ^11^Global Ecology Unit (CREAF), Catalonia, Spain; ^12^Institut Barcelona d'Estudis Internacionals, Barcelona, Spain; ^13^Barcelona Supercomputing Center (BSC), Barcelona, Spain; ^14^Catalan Institution for Research and Advanced Studies (ICREA), Barcelona, Spain; ^15^Centre on Climate Change & Planetary Health and Centre for Mathematical Modelling of Infectious Diseases, London School of Hygiene and Tropical Medicine, London, United Kingdom; ^16^Estudis d'Economia i Empresa, Universitat Oberta de Catalunya, Barcelona, Spain; ^17^Internet Interdisciplinary Institute (IN3), Universitat Oberta de Catalunya, Barcelona, Spain; ^18^Unesco Chair on Food, Culture and Development, Universitat Oberta de Catalunya, Barcelona, Spain; ^19^UNESCO Chair in Life Cycle and Climate Change, ESCI-UPF, Barcelona, Spain; ^20^Department of Law, Faculty of Law, Pompeu Fabra University, Barcelona, Spain; ^21^Institut de Ciència i Tecnologia Ambientals (ICTA), Universitat Autònoma de Barcelona, Barcelona, Spain; ^22^Faculty of Government, University of Chile, Santiago, Chile; ^23^Departamento de Ciencia, Tecnología y Sociedad, Insituto de Filosofía, Spanish National Research Council (IFS-CSIC), Madrid, Spain; ^24^Departamento de Filosofía y Antropología, Universidad de Santiago de Compostela (USC), Santiago, Spain; ^25^Faculty of Psychology and Education Sciences, Universitat Oberta de Catalunya, Barcelona, Spain; ^26^COVID-19 Early Detection, Surveillance and Control Department, Public Health Agency of Barcelona, Barcelona, Spain; ^27^Mariana Arcaya's Research Lab, Massachusetts Institute of Technology Department of Urban Studies and Planning, Cambridge, MA, United States; ^28^Johns Hopkins University - Universitat Pompeu Fabra (JHU-UPF) Public Policy Center, UPF-BSM, Department of Political and Social Sciences, Universitat Pompeu Fabra, Barcelona, Spain; ^29^Research Group on Health Inequalities, Environment, and Employment Conditions (GREDS-EMCONET), Department of Political and Social Sciences, Universitat Pompeu Fabra, Barcelona, Spain; ^30^IMIM Hospital del Mar Medical Research Institute, Barcelona, Spain

**Keywords:** curriculum development, education for sustainable development (EfSD), Master in Science, planetary health education, postgraduate education, sustainable development goals –SDG

## Abstract

Planetary Health has emerged as a new approach to respond to the existential risks that the clime and global environmental crises pose to human societies. As stated by various stakeholders, the challenges involved in Planetary Health are of such magnitude that education must be at the forefront to obtain a meaningful response. Universities and higher education institutions have been specifically called to embed the concept of planetary stewardship in all curricula and train the next generation of researchers and change makers as a matter of urgency. As a response to this call, the Universitat Oberta de Catalunya (UOC), the Universitat Pompeu Fabra (UPF), and the Barcelona Institute for Global Health (ISGlobal) developed the first online and asynchronous Master in Science (MSc) in Planetary Health. The aim of the programme is to train a new generation of academics and professionals who understand the challenges of Planetary Health and have tools to tackle them. This article describes the development of the curriculum of this MSc, presents the main characteristics of the programme and discusses some of the challenges encountered in the development of the programme and its implementation. The design of this MSc was based on: the alignment of the programme with the principles for Planetary Health education with a focus on human health; a multi-, inter-, and trans-disciplinary approach; the urgency to respond to the Anthropocene challenges; and the commitment to the 2030 Agenda. The MSc was recognized as an official degree by the Agency for Quality of the Catalan University System, included in the European Quality Assurance Register for Higher Education, and the Spanish National Academic Coordination body in April 2021 and launched in October 2021. There are currently more than 50 students enrolled in the program coming from a broad range of disciplines and geographic locations. The information presented in this article and the discussion on challenges encountered in developing and implementing the programme can be useful for those working in the development of similar programs.

## Introduction

### Background and rationale for the educational activity innovation

In the last decades, there has been an increasing understanding of the socio-environmental transformations—accelerated by the Anthropocene —and how they pose an existential risk to human societies and other living beings. As a result, several approaches emerged to connect the environment and human health (1, 2) and respond to new threats, such as the climate crisis, biodiversity loss and toxic pollution (1). One of the new approaches, Planetary Health, is based on the comprehension that human health and human civilization depend on flourishing natural systems and their stewardship. This point of view requires unprecedented efforts to deal with complexity and uncertainty, encourage transdisciplinary and urgent action (3).

Similar to other scientific fields (4), the challenges involved in Planetary Health are of such magnitude that education at all levels must be at the forefront to obtain a meaningful response (5, 6). The São Paulo Declaration on Planetary Health, a global call to action from the planetary health community supported by more than 300 signatories, urged to include planetary health education in all curricula levels, from schools to universities (7). The UN report “The Future is Now” has specifically called upon universities and higher education institutions to support the mission of advancing sustainability. This recognizes that the education of the next generation of researchers and change makers is one of the best leverage strategies toward transformations in sustainability (8). Recently, the ‘Our Planet, Our Future” call for action—signed by a large number of Nobel laureates—requested universities and higher education institutions to urgently embed the concept of planetary stewardship in all curricula (9).

In consequence, there is a growing number of initiatives to transform higher education for sustainable health. The Association of Medical Education in Europe (AMEE) has suggested that to reduce CO_2_ emissions and to meet the UN's 2030 Sustainable Development Goals (SDGs), health-related studies must equip undergraduates (and already qualified professionals) with the necessary knowledge, skills, values, competence, and confidence (10). The Global Consortium on Climate and Health Education (GCCHE) surveyed 160 institutions to understand the state of climate-health curricula for health professions. The results showed that educational programmes vary considerably between institutions and that the majority of responders faced relevant challenges when trying to implement curricular changes in their institutions (11). A similar study conducted in Latin-America has shown that universities in this continent have similar drivers and barriers for sustainability change as universities in other geographical contexts (12).

There is growing evidence of new methodologies and approaches to include Planetary Health in health curricula (13–15). Among the different types of curricula, postgraduate education has received little attention and yet it offers a unique opportunity to train already qualified professionals from different disciplines to work multi- (drawing on knowledge from different disciplines but remaining within the boundaries of those fields), inter- (analyzing, synthesizing, and harmonizing links between disciplines into a coordinated and coherent whole), and trans-disciplinary (using a shared conceptual framework drawing together new disciplinary-specific theories, concepts, and approaches to address common problems) (16). In the GCCHE study cited above, only one institution reported having a master's or certificate programme in climate and health. Its respondents reported that it had been virtually impossible to develop new courses on climate and health in public health master's programmes due to the already high course load (11). To contribute to the development of Planetary Health education at the postgraduate level, the *Universitat Oberta de Catalunya (UOC*), the *Universitat Pompeu Fabra (UPF)*, and the Barcelona Institute for Global Health (ISGlobal) have developed an online Master in Science (MSc) in Planetary Health. It was launched in October 2021 and will be fully implemented in March 2023.

This article describes the main characteristics of this new programme and discusses some of the challenges we are currently facing.

### Overview of the master in science in planetary health

**Figure 1:**
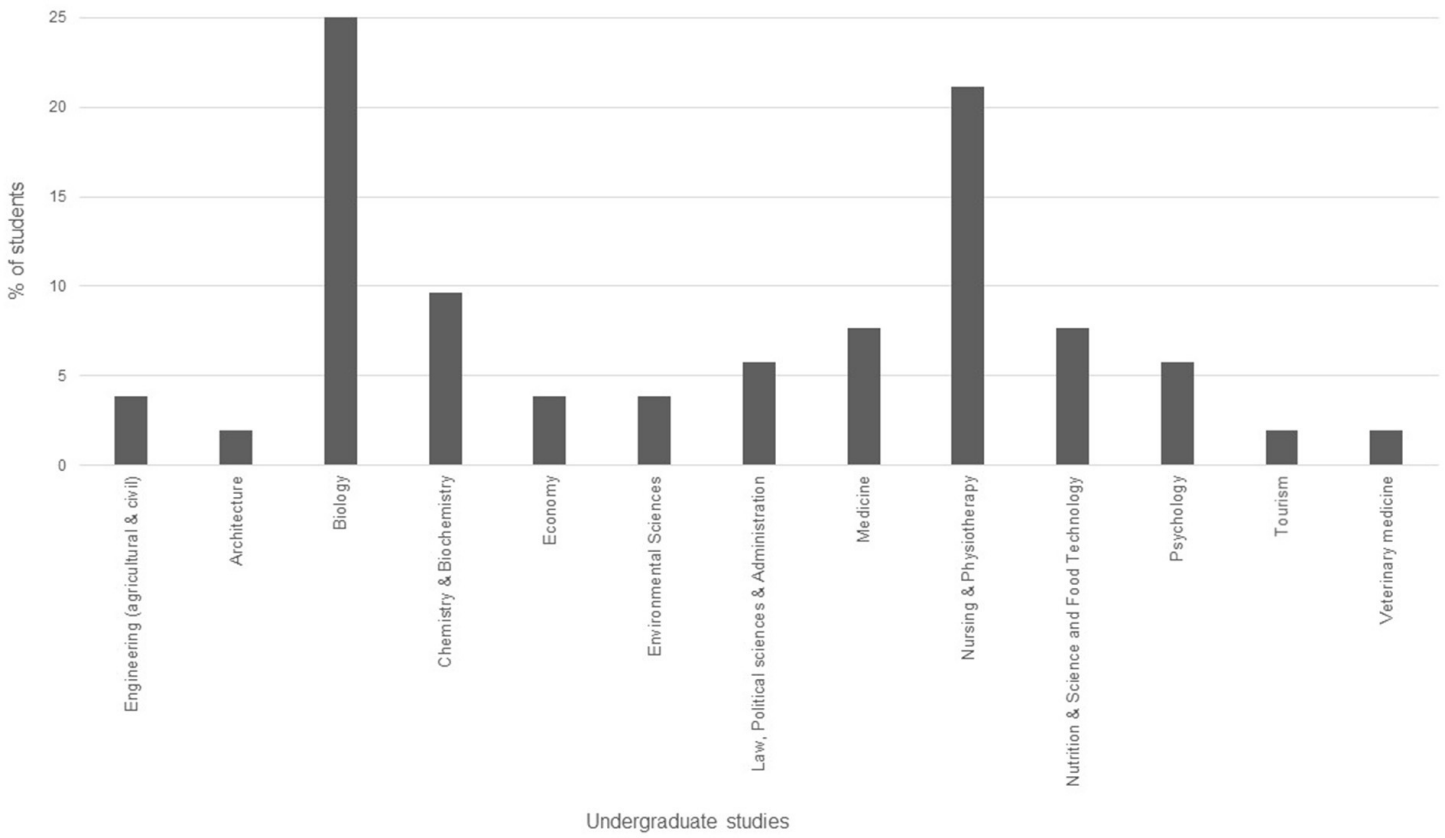
Description of the academic backgrounds of the first cohort of the MSc in Planetary Health (*n* = 55).

The MSc in Planetary Health (UOC-UPF-ISGLOBAL) is a fully online and asynchronous programme of 60 ECTS credits (European Credit Transfer and Accumulation System, 1 ECTS is equivalent to 25 h). In its first edition, the MSc in Planetary Health has been offered in Spanish and Catalan. The academic entry requirements encompass undergraduate studies from a broad range of disciplines, including health sciences, natural sciences, political sciences, economical sciences, sociology, law, and engineering. The first cohort of students (first term October 2021) captures this multidisciplinary profile (see Figure 1). This fist cohort was integrated by 55 students (75% females, 25% males), 87% of them were from Spain, 7% from other European countries and 5 % from Latin America.

The overall aim of the programme is to provide a multi-, inter- and transdisciplinary academic syllabus, as well as applied education on Planetary Health to train, promote, and empower a new generation of academics and professionals. They will be able to contribute to understanding the full complexity of the challenges related to Planetary Health and well-being; from which they will develop and find solutions and strategies to tackle these challenges. To achieve this aim, the design of this MSc degree was based on a set of general criteria: (i) the programme content is aligned with the principles for Planetary Health education (17) with a focus on human health; (ii) it includes the essential multi-, inter-, and trans-disciplinary aspects of Planetary Health challenges; (iii) it transmits a sense of urgency as a consistent attitude, considering the timeframe of the challenges that involve climate and the Earth's natural systems; and (iv) it is aimed to create an impact and it is committed to the 2030 Agenda.

This MSc programme was recognized as an official degree by the Spanish academic system on April 2021. Official MSc allow the enrolment in PhD programs and therefore are a way to promote research in a given field. Official degrees are subjected to a thorough evaluation process: this programme was evaluated and approved by the Agency for Quality of the Catalan University System (AQU Catalunya), included in the European Quality Assurance Register for Higher Education (EQAR), and the Spanish National Academic Coordination body (*Consejo de Universidades*). The programme was also reviewed and supported by an international advisory committee, which involved researches and academics working in areas relevant to Planetary Health.

## Methods

### Definition of the programme

**Figure 2:**
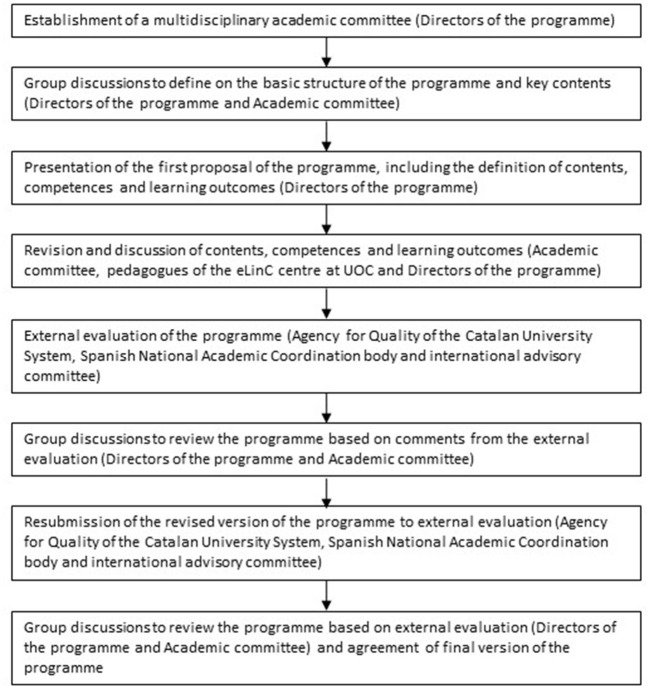
Steps followed to define the programme and the actors involved in each step.

The definition of the programme was a collaborative process in which several actors were involved. Throughout several group discussions, the direction of the programme and the academic committee (https://estudios.uoc.edu/es/masters-universitarios/salud-planetaria/profesorado) agreed on the structure and the contents of the programme (presented in the following section). The academic committee included scholars working in multiple disciplines, such as public health, environmental epidemiology, climate sciences, ecology, political ecology and political economy. Specialist in pedagogy and educational methods from the eLearning Innovation Center (eLinC) centre at UOC (https://www.uoc.edu/portal/en/elearning-innovation-center/coneix/index.html), contributed in the definition of the specific learning outcomes and identify the best methodologies to achieve them. Figure 2 describes the steps followed to prepare the final version the programme and the actors involved in each step of the process.

### Structure and coherence of the programme

The MSc in Planetary Health (UOC-UPF-ISGLOBAL) is organized in twelve modules of 5 ECTS each (equivalent to 125 h) and structured in three thematic blocks (see Table 1). The first block (three modules) provides the general context and the necessary methodologies for understanding and responding to the Planetary Health challenges of the Anthropocene. It also sets the bases for an effective multilevel global governance. The second block (which includes six modules) focuses on issues identified as key challenges for Planetary Health: food systems, change in land use and loss of biodiversity, water resources, global pollution, urbanization, healthy and sustainable cities, and the climate emergency. The main objective is to develop the student's critical understanding of the origins and causes of these issues and its effects on human health to devise and design potential solutions, as well as to evaluate possible problems and risks when implementing them. In that sense, all modules from block 2 are solution oriented and are designed according to this scheme: (i) description of the challenge, (ii) potential solutions to the challenge, and (iii) evaluation of the possible problems and risks when implementing them. The third block (which includes three modules) integrates and applies the concepts from the two previous blocks. It includes a module with strategies that promote transformative changes to address the challenges of Planetary Health, including the role of citizen action and social movements; a second module to introduce research on Planetary Health and familiarize students with a broad range of research areas and disciplinary approaches; and a third module, focused on the master's thesis. The specific competences and their distribution thought the modules are presented in Table 2. Further details on each module can be found in the Supplementary material (Supplementary material: Curriculum of the MSc in Planetary Health).

**Table 1:** Organization of the MSc in Planetary Health in relation to the domains defined in the Planetary Health educational framework (18).

**Tematic blocks**	**Modules**	**Main domain covered**
Block 1: concepts and methods	1. Planetary Health, the response to the challenges of the Anthropocene	The anthropocene and health
	2. Interdisciplinary approaches to Planetary Health	Systems thinking and complexity
	3. Global and multilevel governance in planetary health	Equity and social justice
Block 2: challenges in planetary health	4. Sustainable food systems and healthy diets	Understanding interconnection within nature
	5. Land use change, biodiversity loss and human health	
	6. Water resources and Planetary Health	
	7. Global pollution and health	
	8. Urbanization and healthy and sustainable cities	
	9. Climate change and health	
Block 3: application of knowledge	10. Integrative solutions and transformative changes	Movement building and systems change
	11. Planetary Health Research: From the hypotheses to the research protocol	
	12. Master's thesis	

**Table 2:** Distribution of competences and skills across the modules of the programme.

**Competences and skills**	**Modules**											
	**1**	**2**	**3**	**4**	**5**	**6**	**7**	**8**	**9**	**10**	**11**	**12**
**Basic competences and skills**												
CB6 – Gain and understand knowledge that forms the basis or an opportunity for original thinking in the development and/or application of ideas, typically in a research context	x		x								x	x
CB7 – Capacity to apply the acquired knowledge and capacity for problem-solving in new or relatively unknown environments within broader (or multidisciplinary) contexts related to the field of studies.			x		x	x						x
CB8 – Capacity to integrate knowledge and tackle the complexity of formulating judgements based on incomplete or limited information, taking due consideration of the social and ethical responsibilities involved in applying knowledge and making judgements	x			x	x	x	x	x	x			x
CB9 – Capacity to communicate conclusions and the knowledge and grounds on which they have been reached to specialist and non-specialist audiences in a clear and unambiguous manner				x				x				x
CB10 – Learning skills that enable ongoing self-directed and independent learning				x	x	x	x	x				
**General competences and skills**												
CG1 – Capacity to search for, analyze, assess, and use information provided to make decisions in complex situations	x	x			x	x	x					x
CG2 – Capacity to work in interdisciplinary teams to attain shared goals in relation to planetary health		x			x		x			x		
CG3 – Capacity to apply creative thinking to contribute improvements or solutions in areas and situations of varied complexity in relation to planetary health			x						x	x		x
CG4 – Capacity to resolve complex situations in a feasible, sustainable way, by analyzing their components, finding alternatives, reaching consensus on their application and assessing the results of their implementation												
**Transversal competences and skills**												
CT1 – Capacity to act in an honest, ethical, sustainable, socially responsible and respectful way considering human rights and diversity, both in academic and professional practice, and design solutions to improve these practices		x	x							x	x	x
**Specific competences and skills**												
CE1 – Analyze the interaction between human health and the Earth's natural systems, using complex theoretical and conceptual models that relate the factors that promote environmental change, their effects on health, and allow for possible solutions to guarantee health in a sustainable way	x			x	x	x	x	x	x	x		
CE2 – Design research projects and interventions, applying and integrating advanced knowledge on socioeconomic, political and / or cultural factors that interact affecting human health and promoting the degradation of natural systems				x	x			x			x	x
CE3 – Critically interpret, synthesize and integrate the results of quantitative and qualitative analysis from research in the main scientific disciplines related to Planetary Health (health sciences, natural and climate sciences, social sciences and economics)		x					x	x			x	x
CE4 – Select and apply advanced methodologies and resources from different scientific disciplines in the field of Planetary Health to strategically solve complex situations and problems	x										x	x
CE5 – Mastering the language and communicative conventions of the disciplinary fields of Planetary Health in order to act as an interlocutor in the professional field, formulating arguments and transmitting results and ideas in a clear and unambiguous way		x	x					x	x			x
CE6 – Implement with initiative and autonomy research projects or interventions in the field of Planetary Health, integrating a multidisciplinary vision, transferring the main results to the actors involved											x	x
CE7 – Critically evaluate and apply innovative proposals for solutions for the prevention, promotion and management of health with a multidisciplinary approach, taking into account environmental sustainability and equity				x	x	x	x	x	x	x	x	x
CE8 – Formulate predictions about the evolution of health problems based on changes in natural systems, through innovative and multidisciplinary approaches that consider socioeconomic, political and / or cultural factors	x	x	x	x					x			x

To make the MSc in Planetary Health a coherent programme, its structure and contents were aligned with the Planetary Health educational framework and the SDGs. Moreover, the competences and learning outcomes (see Table 1) cover the majority of overarching principles for Planetary Health education (17).

The MSc encompasses the five domains (see Table 1) proposed by the Planetary Health educational framework (18). The domains on “*The Anthropocene and Health*, “*Systems Thinking and Complexity*” and “*Equity and Social Justice*” are mainly addressed on the first thematic block of the programme. The need to understand human beings and natural systems as interconnected entities (“*Understanding Interconnection within Nature*” domain) is a cross-cutting theme in all subjects of the degree, and it is especially relevant in subjects of the second thematic block. In this second block, students have to reflect on the root causes of the global environmental crisis, leading them to recognize our disconnection from nature. The domain on “*Movement Building and Systems Change*” is mainly covered in the third thematic block of the master; it is oriented towards integrative solutions and transformative changes. In addition, the five domains are—up to certain level—incorporated in all modules, providing additional coherence to each module and throughout the programme.

The SDGs framework is embedded in the conceptualization of the programme. It is present in the different learning and teaching materials, as well as in the activities. For example, in the module “Planetary Health, the Response to Anthropocene Challenges,” students are asked to explore how SDGs could operationalize the concept of Planetary Health. This exercise aims to clarify that sustained improvements in human health and well-being are linked to the preservation of key natural systems, and supported by good governance and appropriate policies (3). However, the 2030 Agenda is also approached from a critical point of view through the programme discussing the need of a more ambitious and urgent framework to deal with the climate and ecological crises and acknowledging some of the criticisms the Agenda received (19). The module “Global and multilevel governance in Planetary Health” integrates this critical vision. by shedding light on the difficulties and governance challenges associated with the implementation of the most relevant SDGs for Planetary Health.

### Learning environment and pedagogical format

The degree has been implemented following the UOC asynchronous online educational model (20), which is underpinned by two principles: learning by doing and autonomous learning. Following this model, each module of the MSc in Planetary Health is organized around the resolution (or response) to a number of challenges. The challenges are inspired by real contexts of the different disciplinary areas and are oriented to develop defined personal and professional skills (see Supplementary material). This is achieved by asking the student to complete a series of activities and/or prepare deliverables for each of the challenges. The evaluation of the modules is based on the continuous assessment of such activities and deliverables during the term.

Each of the challenges includes: the approach to the addressed issue, the description of activities to develop key skills, and the learning resources and tools to complete the activities and/or prepare the deliverables. Learning resources are found in the virtual classroom in a visual way. For each resource, guidance on how to use it in the context of the activity and the expected amount of time required to complete each challenge is provided. The learning resources available have a wide range of formats (websites, video, audio, texts, or digital tools); and include both teaching resources prepared by faculty members and external resources (academic papers, book chapters, scientific reports, recorded academic conference presentations, infographics, and documentaries, among others).

During the autonomous learning process, students are supported by the faculty. In the UOC educational model, there are two faculty roles: coordinating professors and course instructors. Coordinating professors design the content of the module, coordinate and supervise the team of course instructors, and supervise the evaluation process. Course instructors are in close contact with the students by introducing the activities for each challenge, promoting participation in the virtual classroom, solving specific questions, providing feedback, and evaluating the activities and deliverables.

The multi-, inter-, and trans-disciplinary aspects of the programme are central to Planetary Health (3) and a hallmark of the MSc in Planetary Health. The syllabus has been developed by a multidisciplinary academic committee, including experts on public health, environmental epidemiology, climate change, political ecology and geography, international law, social sciences, economics, and ecology. All modules are regularly reviewed and discussed by the academic committee, ensuring that diverse approaches and views are adequately considered.

In the Supplementary material, we present an example of how the UOC educational model is applied to one of the modules of the MSc in Planetary Health (Supplementary material: Example of the implementation of UOC educational model in the MSc: Module 1 Planetary Health: The Response to Anthropocene Challenges).

### Anticipated career trajectories

The programme was planned to promote a new generation of professionals and academics able to apply a Planetary Health approach in their careers. Specifically, the programme aims to provide capabilities in research, education and professional work.

As official degree by the Spanish academic system, our programme incorporates a strong focus on research as the source of new knowledge. All modules strengthen the importance of knowledge generation as part of the solution to the climate and environmental crises. Also, the programme provides a research path for those interested in pursuing a PhD programme. Thus, following a career as researcher is one of the possible trajectories for our graduates.

Graduates are also anticipated to develop careers in education and several of our registered MSc students are already teaching in high-schools and universities. As commented above, inclusion of the Planetary Heath in the curricula of all programs and faculties has been also stated as a priority by various stakeholders (5–9). Though our MSc does not provide training in education methods, it is proving useful both for those in education to incorporate planetary health contents in their programs as well as to acquire training in the adoption of online education methods.

Finally, and consistent with the consequential and solution-oriented spirit of planetary health, our MSc is also strongly oriented to support career opportunities for professional working in public and private sectors directly dedicated to health and the management of natural resources, urbanization, or transport among others. For instance in (i) international agencies and / or non-governmental organizations (NGOs) that work in the field of health, environmental preservation and/or sustainable development, (ii) health services that seek to integrate aspects of environmental sustainability in health care and management, (iii) public administration that works in the development and / or implementation of land management plans, town planning or energy, among others or (iv) in the sustainability and corporate social responsibility departments of companies related to health and the environment.

## Discussion

This article describes the development of a multidisciplinary online master degree on Planetary Health, showing its structural consistency with the Planetary Health educational framework (18) and the UN's Agenda 2030. In concordance with the urgency that the climate and ecological crises request (21), the new degree has received the support of the national accreditation bodies and has attracted a first cohort of students with a wide range of academic backgrounds in a very short period of time. We have integrated the guiding criteria of complexity, multidisciplinary, and urgency (3) to develop a feasible and innovative programme for postgraduate education in Planetary Health, which is also consistent with a wider set of cross-cutting principles for Planetary Health education (17).

Planetary Health involves a paradigm shift compared to global public health. Planetary Health integrates human health with the health of other species and Earth's natural systems, something that implies a broader ethical perspective to explicitly account for the value of future human and other species generations. Planetary Health also extends the global public health predicament of social sciences approach in dealing with health inequalities and equity to the understanding of the interactions between social systems and ecosystems and to deliver solutions to protect and restore the natural systems on which human health depends. Planetary Health shares this paradigm shift with other approaches. Busse et al. (1) have analysed some of the health approaches that connect the health of ecosystems, other living organisms and humans, including occupational and environmental health; political ecology of health; environmental justice; eco-health; One Health; and ecological public health. The proliferation of approaches emerging from different disciplinary fields, can lead to confusion due to overlaps in concepts and terminology. As a result of the new approaches, there are numerous initiatives to align postgraduate education in the health sector with the challenges of the climate crisis and the Anthropocene. For instance, the Faculty of Public Health and Training Programme Directors—from the 13 public schools specialized in health across the UK—have recently reported an initiative to strengthen sustainable development in public health consultant education (22). Postgraduate education in One Health has already come a long way with a large number of master programmes, which either highlight One Health in the programme's name or include it as a feature component (23). In contrast, postgraduate education initiatives focusing on Planetary Health are less common. To our knowledge there are no other master degrees on Planetary Health. However, we are aware of other universities planning to launch masters on Planetary Health soon and the Stanford University and the London School of Hygiene & Tropical Medicine (LSHTM) have recently launched a Planetary Health Postdoctoral Fellowship programme (24). Moreover, It is very likely that many schools of public health are currently developing specific postgraduate modules on Planetary Health like the ones in the University of Toronto Dalla Lana School of Public Health (25) and the UPF (26).

Beyond the described initiatives, to our knowledge, our programme is one of the first MSc degrees fully devoted to Planetary Health. A relevant innovative component is its online and asynchronous methodology, responding to a call by young academics to expand online training for Planetary Health, which can contribute to boost access to high-quality education programmes for global audiences (5) and foster education at different stages of the professional career. The MSc is available in Spanish, offering an opportunity to expand Planetary Health education to Spanish-speaking countries. English version is currently being considered.

A major consequence of the global and complex nature of Planetary Health challenges is the need of close collaborations across different disciplines; the approaches towards such collaboration are diverse and they can be multi-, inter-, and trans-disciplinary. The need to adopt these approaches is based on the assumption that the Anthropocene's challenges are so complex that their thorough examination and solution requires different scientific disciplines to work in alliance with each other, something that requires the combination of efforts and knowledge from different disciplines, including but not limited to medicine, biology, climate science, economics, political science, law, humanities, culture or technology (27). To achieve this in our programme, we have drawn from our own experience with the Planetary Well-being Initiative, (15) as well as from previous calls for a cross-disciplinary collaboration in Planetary Health (3, 18, 20).

Postgraduate education provides a unique opportunity for students with different disciplinary backgrounds to meet in inter-professional education programmes (28). The importance of multidisciplinary and inter-professional education has been largely recognized in the postgraduate education of public health and global health (28, 29) and we have built on this tradition.

The multi-, inter-, and trans-disciplinary approach in the educational programme allows students to share a common set of conceptual models and to be exposed to a wide range of methods. This will strengthen their capacity to work alongside colleagues from other disciplines throughout their careers. The adoption of a multi-, inter-, and trans-disciplinary approach (16) has several implications, such as the need of a faculty with a diverse background in the disciplines involved in the *corpus* of Planetary Health. In the current stage of the MSc in Planetary Health, the diversity of disciplinary backgrounds is well-stablished in the team of course instructors, in the academic committee that supervised the design of the programme and the modules, and in the external advisory committee who played a key role during the accreditation phase. However, the adoption of this approach also involves some relevant challenges. One of them is to provide students with additional contents to be able to follow those topics that are far from their own academic background. Another very important challenge is to guarantee that all students receive an advanced education level with a strong focus on research (instead of an introductory education to a wide subject's thematic range). So, adopting a wide multi-, inter-, and transdisciplinary approach in a master degree involves a tension between the large diversity of theories, concepts and methods and the degree of deepness that a master level requires. This tension was already noted by the evaluators in the accreditation phase. To address these difficult issues, we are conducting targeted actions that require close monitoring and evaluation of the MSc programme.

A potential limitation of the MSc at its current stage is the limited presence of local knowledge (e.g., traditional indigenous knowledge and local ecological knowledge) in the curricula. The inclusion of local knowledge and ideas to navigate sustainability locally is in general poorly addressed within higher education (30). However, we believe that including this content in the curricula will emphasize the importance of the human-nature relationship in other worldviews, as well as recognize the relevance of traditional knowledge in monitoring changes in nature and in providing examples of successful adaptation to these changes (31–33). To address this issue, we will explore collaborations with indigenous scholars to further integrate local knowledge in the MSc.

Universities and higher education institutions have a pivotal role in Planetary Health: (3) they have been called to urgently embed the concept of planetary stewardship in all curricula (9). In our strategy to include Planetary Health in the university, we have maintained a sense of urgency as a core principle (15). However, dealing with the complexity of university bureaucracy and complying with the necessary academic accreditation standards requires time and resources. For us, it was key to have a strong support from the participating institutions. This allowed us to develop the programme, obtain the approval of the Spanish academic system, elaborate and implement the initial learning resources, and recruit the first cohort of students in about 2 years. We lacked the reference of any previous MSc in Planetary Health, which made our task more challenging.

The motivation letters of our students show a strong desire for educational programmes that provide them with academic training on relevant scientific knowledge and training to become planetary stewards. We hope that our experience is useful and inspiring for other institutions to create similar programmes, which would result in further opportunities for collaboration and mutual learning. Our goal is to generate a critical mass of professionals with Planetary Health knowledge and values, willing and able to coordinate inter-professional teams and to work effectively with cross-sector stakeholders to solve today's and tomorrow's pressing challenges.

## Data availability statement

The original contributions presented in the study are included in the article/Supplementary material, further inquiries can be directed to the corresponding author/s.

## Ethics statement

Ethical review and approval was not required for this study in accordance with the local legislation and institutional requirements. Written informed consent from the academic committee participants was not required to participate in this study in accordance with the national legislation and the institutional requirements.

## Author contributions

JA and CO'C-G are the directors of the programme, conceptualized the curriculum, the paper, and wrote the original daft. AM contributed to the writing of the original daft and prepared the figures. MB-P, JJ, RL, CT, and CZ contributed to the conceptualized the curriculum and reviewed and edited the original draft of the article. EC-S, PD, CG, AG-J, MG, OG, HM, FM, LM, GN, AN, IR-M, NS-V, and MT-M reviewed and edited the original draft of the article. All authors contributed to the article and approved the submitted version.

## Conflict of interest

The authors declare that the research was conducted in the absence of any commercial or financial relationships that could be construed as a potential conflict of interest.

## Publisher's note

All claims expressed in this article are solely those of the authors and do not necessarily represent those of their affiliated organizations, or those of the publisher, the editors and the reviewers. Any product that may be evaluated in this article, or claim that may be made by its manufacturer, is not guaranteed or endorsed by the publisher.
