# Supplementary material for: Responding to the need of postgraduate education for Planetary Health: Development of an online Master's Degree
Source: Front Public Health. 2022 Oct 26;10:969065. doi: 10.3389/fpubh.2022.969065 (PMC9643718; doi:10.3389/fpubh.2022.969065)
Supplement: Supplementary file 1 [file Presentation_1.pdf]

## Supplementary material

# Responding to the need of postgraduate education for Planetary Health: The Development of an on-line Master's Degree.

Cristina O'Callaghan-Gordo[1,2,3,4*†], Ariadna Moreno[2], Marina Bosque-Prous[1], Enrique Castro-Sanchez[5,6], Payam Dadvand[2,3,4], Carlos A. Faerron Guzmán[1,2,7,8], Ana García-Juanatey[1,9], Mireia Gascon[1,2,3,4], Oriol Grau[10,11], Jacint Jordana[3,12], Rachel Lowe[13,14,15], Hug March[16,17], F. Xavier Medina[1,18], Lela Mélon[1,19,20], Grettel Navas[1,21,22], Andrea Núñez Casal[1,23,24], Isabel Ruiz-Mallén[17,25], Nacho Sánchez-Valdivia[1,26], Cathryn Tonne[2,3,4], Margarita Triguero-Mas[1,27], Christos Zografos[28,29] and Josep M. Antó[2,3,4,30†]

[†]These authors have contributed equally to this work

[*]Corresponding author: Cristina O'Callaghan-Gordo. Email adress: co_callaghangoc.edu. Phone number: +34 690011723

[1]Faculty of Health Sciences, Universitat Oberta de Catalunya, Barcelona, Spain, [2]ISGlobal, Barcelona, Spain, [3]Universitat Pompeu Fabra (UPF), Barcelona, Spain, [4]CIBER Epidemiología y Salud Pública (CIBERESP), Madrid, Spain, [5]College of Nursing, Midwifery and Healthcare, University of West London, Brentford, United Kingdom, [6]Health Protection Research Unit in Healthcare Associated Infection and Antimicrobial Resistance, Imperial College London, London, United Kingdom, [7]Graduate School, University of Maryland, Baltimore, MD, United States, [8]Planetary Health Alliance, Harvard T.H. Chan School of Public Health, Boston, MA, United States, [9]CEI International Affairs, Barcelona, Spain, [10]Plants and Ecosystems, University of Antwerpen, Antwerpen, Belgium, [11]Global Ecology Unit (CREAF), Catalonia, Spain, [12]Institut Barcelona d'Estudis Internacionals, Barcelona, Spain, [13]Barcelona Supercomputing Center (BSC), Barcelona, Spain, [14]Catalan Institution for Research and Advanced Studies (ICREA), Barcelona, Spain, [15]Centre on Climate Change & Planetary Health and Centre for Mathematical Modelling of Infectious Diseases, London School of Hygiene and Tropical Medicine, London, United Kingdom, [16]Estudis d'Economia i Empresa, Universitat Oberta de Catalunya, Barcelona, Spain, [17]Internet Interdisciplinary Institute (IN3), Universitat Oberta de Catalunya, Barcelona, Spain, [18]Unesco Chair on Food, Culture and Development, Universitat Oberta de Catalunya, Barcelona, Spain, [19]UNESCO Chair in Life Cycle and Climate Change, ESCI-UPF, Barcelona, Spain, [20]Department of Law, Faculty of Law, Pompeu Fabra University, Barcelona, Spain, [21]Institut de Ciència i Tecnologia Ambientals (ICTA), Universitat Autònoma de Barcelona, Spain, [22]Faculty of Government, University of Chile, Santiago, Chile, [23]Departamento de Ciencia, Tecnología y Sociedad, Insituto de Filosofía, Spanish National Research Council (IFS-CSIC), Madrid, Spain, [24]Departamento de Filosofía y Antropología, Universidad de Santiago de Compostela (USC), Santiago, Spain, [25]Faculty of Psychology and Education Sciences, Universitat Oberta de Catalunya, Barcelona, Spain, [26]COVID-19 Early Detection, Surveillance and Control Department, Public Health Agency of Barcelona, Barcelona, Spain, [27]Mariana Arcaya's Research Lab, Massachusetts Institute of Technology Department of Urban Studies and Planning, Cambridge, MA, United States, [28]Johns Hopkins University - Universitat Pompeu Fabra (JHU-UPF) Public Policy Center, UPF-BSM, Department of Political and Social Sciences, Universitat Pompeu Fabra, Barcelona, Spain, [29]Research Group on Health Inequalities, Environment, and Employment Conditions (GREDS-EMCONET), Department of Political and Social Sciences, Universitat Pompeu Fabra, Barcelona, Spain, [30]IMIM Hospital del Mar Medical Research Institute, Barcelona, Spain

**Table of contents**

**Curriculum of the MSc in Planetary Health: .......... 3**

Module 1. Planetary Health, the Response to the Anthropocene Challenges .......... 3

Module 2. Interdisciplinary Approaches for Planetary Health .......... 3

Module 3. Global and multilevel governance in Planetary Health .......... 4

Module 4. Sustainable food systems and healthy diets .......... 4

Module 5. Change in land use, loss of biodiversity and human health .......... 5

Module 6. Water Resources and Planetary Health .......... 6

Module 7. Global pollution and health .......... 6

Module 8. Urbanization and healthy and sustainable cities .......... 7

Module 9. Climate change and health .......... 8

Module 10. Integrative Solutions and Transformative Changes .......... 9

Module 11. Planetary Health Research: From Hypotheses to Research Protocol .......... 9

Module 12. Master's thesis .......... 10

**Example of the implementation of UOC educational model in the MSc: Module 1 ¨Planetary Health: The Response to Anthropocene Challenges" .......... 11**

**Curriculum of the MSc in Planetary Health:**

## MODULE 1. Planetary Health, the Response to the Anthropocene Challenges

**Content.** The Anthropocene is characterized by great improvements in human development, as well as by significant degradation of ecosystems. The general objective of this module is to analyze the changes that the Anthropocene entails in natural systems and the main challenges that these changes pose for health. This module will also introduce the main governance strategies for development and the implementation of responses that will be addressed in detail in other modules of the master's degree, with special reference to the United Nations 2030 Agenda.

- Current changes in Earth's global systems and their impact on human health.
- Social, economic and political determinants of anthropogenic change in natural systems and their influence on human health.
- Key human health problems related to changes in natural systems.
- Planetary boundaries.
- Conceptual, research and implementation challenges in Planetary Health.
- 2030 Sustainable Development Goals.

**Learning outcomes.** At the end of this course students will be able to:

- Critically analyze the main changes in Earth's natural systems that characterize the Anthropocene, and explain the complex dynamics and interactions of these changes in the context of the most recent scientific research.
- Interpret complex health problems under the lens of Planetary Health.
- Integrate advanced knowledge on the main social determinants of anthropogenic change in natural systems and analyze its impact on human health.
- Propose solutions to complex problems derived from the interaction between human health and natural resources in the context of Planetary Health approaches.

## MODULE 2. Interdisciplinary Approaches for Planetary Health

**Content.** The challenges of Planetary Health result from complex interactions between natural, economic and social systems. To understand and analyze the origin of these problems, as well as to propose innovative solutions, knowledge with a multidisciplinary and transdisciplinary approach is required. The objective of this module is to provide students with the knowledge and tools necessary to interact with other disciplines, as well as to incorporate a multidisciplinary vision into their final master's work, through knowledge of the main approaches, methods and resources of the disciplines. most relevant to Planetary Health. The objective of this module is for students to be able to (i) identify and understand the role of the main scientific and academic disciplines relevant to Planetary Health, as well as their main theoretical approaches and methodologies; (ii) develop a multidisciplinary understanding of information systems and evaluation methods for programs and policies related to sustainability and health; and (iii) apply interdisciplinary and transdisciplinary approaches to respond to complex challenges in the field of planetary health.

- Multi-, inter- and trans-disciplinarity.
- Major Scientific Disciplines for Planetary Health: Approaches, and Resources.
- Interdisciplinary models and methodologies for meeting Planetary Health challenges
    - Complex systems.
    - Feedback mechanisms.
    - Points of no return.
    - Integrated assessment models (IAM): International Futures (IFs), Shared Socioeconomic Pathways (SSPs).
    - Frameworks for developing environmental health indicators.

**Learning outcomes.** At the end of this course students will be able to:

- Analyze the main international multidisciplinary scientific networks in relation to the interactions between health and natural resources.
- Manage the information systems and evaluation methods of different disciplines, relevant to Planetary Health.
- Critically interpret the results of theoretical models and methodologies of the main scientific disciplines relevant to Planetary Health.
- Characterize the challenges derived from complexity, uncertainty and ambiguity with a multidisciplinary approach.
- Apply multi-, inter- and trans-disciplinary approaches to solve Planetary Health problems, integrating collaboration between researchers and specialized professionals.
- Analyze and assess the importance, need and challenges of multi-, inter and trans-disciplinary in the context of the Anthropocene and the 2030 Agenda.

## MODULE 3. Global and multilevel governance in Planetary Health

**Content.** Governance constitutes one of the key axes to face planetary health challenges. Improving governance for planetary health requires action at the global, national, and subnational levels. The management of world politics in the 21st century is complex: we find more and more actors, more institutions, more formal and informal rules and processes, more conflicting interests and interdependencies. The term global governance synthetically characterizes the complex system of governance at the global level that is emerging, with all kinds of global actors, public and private, which is very different from what we have seen in the past. This module aims to facilitate the understanding of these dynamics, discussing various theoretical frameworks on the structures of global governance, and the dynamics of multilevel policies. The course is particularly focused on the areas most closely related to the governance of planetary health. In this sense, special attention will be paid to the strategies related to planetary health of organizations such as the UN, the WHO and the EU.

- Actors and institutions in the global governance of Planetary Health.
- Theories of global governance: implications for Planetary Health.
- Public policies and multilevel government facing the challenges of Planetary Health.
- Problems and challenges of the global governance of Planetary Health.
  - Complex regimes and planetary health.
  - Global governance of risk and health policies.
  - Challenges for the meta-governance of planetary health.

**Learning outcomes.** At the end of this course students will be able to:

- Examine the basic structures of global governance and differentiate their most common configurations related to the health field.
- Break down the key elements of multilevel governance related to health (coordination structures, decision-making, policy instruments, problem solving).
- Compare multi-level governance structures in planetary health and wellness and determine their links to global governance.
- Critically evaluate coordinated action to respond to the health impacts of the global environmental crisis.
- Make judgments about the application of the ethical principles that guide professional practice in complex situations.

## MODULE 4. Sustainable food systems and healthy diets

**Content.** The sustainability of the global food system is an urgent challenge to ensure the health and quality of life of human beings and the planet in the medium and long term. Providing a healthy diet of sufficient nutritional quality for the entire population without overexploiting the different ecosystems and guaranteeing food security and sustainability, is one of the main challenges of Planetary Health. Currently, the distribution of food at the global level is very unequal and is related to high prevalences of undernutrition and malnutrition, which entail significant global disease burdens. When planning the necessary changes in the food system, one must take into account the complex systemic interactions from the local to the global scale and identify the global limits within which world food production must remain

to safeguard the biophysical processes that sustain a stable earth system. Different strategies both at the production and consumption levels can be implemented, such as dietary changes towards healthy diets, technological and managerial changes in food production, or the reduction of food loss and waste. The objective of this module is for students to be able to analyze the problems posed by food systems in terms of environmental sustainability and human health, and to design and evaluate proposals for new culturally acceptable and economically accessible food models that guarantee the maintenance of the human health and long-term sustainability.

- Healthy and sustainable diet (FAO-WHO): nutritional, environmental, cultural and economic aspects.
- Current trends and projections in the evolution of food production.
- Environmental risks of the intensive food production system.
- Risks to human health of the intensive food production system.
    - Inequality in food distribution.
    - Malnutrition and malnutrition: nutritional deficiencies, overweight and obesity.
    - Morbidity and mortality.
- Strategies to transform food systems into healthy and sustainable.
    - Integrated strategies for healthy eating and sustainable food production globally.
    - Changes in the food consumption model.
    - Food sovereignty, global agreements and sustainable food policies.
    - Food communication and food consumption habits.

**Learning outcomes.** At the end of this course students will be able to:

- Discuss the current challenges of food systems in. relation to human health and sustainability and their complex interrelationships.
- Critically analyze the environmental and health risks associated with the current food production and consumption system, considering the cultural and socioeconomic differences in each territory.
- Critically assess strategies to promote sustainable food systems and predict their impact on health and the environment.
- Design actions that promote a change in the food system towards sustainability, guaranteeing the health of the population and considering the specificities of each territory.

**MODULE 5. Change in land use, loss of biodiversity and human health**

**Content.** More than a quarter of natural habitats have been converted to land for other uses, such as agricultural or industrial areas, and this trend continues to rise in certain regions, especially in the tropics and subtropics. Extensive agriculture and industrial uses of the soil favor its erosion and promote desertification. These processes put human health at risk, food security, and contribute to climate change through the loss of carbon reservoirs. Additionally, the change in land use has a direct effect on the reduction of biodiversity, which has accelerated in the last 40 years, and is currently between 10 and 100 times higher than that observed in the last 10 million years. The loss of biodiversity puts human health at risk through various means, including (i) facilitating the appearance of new emerging diseases, by affecting the control mechanisms of healthy ecosystems to regulate the appearance of new pathogens, (ii) the impact on food security, by reducing the number of pollinators, on which more than 75% of crops depend, and by reducing agrobiodiversity, which favors a better response capacity against pests; (iii) the loss of a main source of obtaining medicines; (iv) the reduction of immune function, by reducing diversity in the human microbiota; and (v) the loss of the beneficial effects of contact with nature at the welfare level. The main objective of this module is to understand the factors that drive the conversion of natural habitats and the loss of biodiversity, analyze the effects that these events have on human health, and evaluate the importance of conservation, restoration and sustainable management policies for the ecosystems and adapt them as tools to preserve health.

- Global and regional trends in the disappearance of natural habitats and driving mechanisms of land use change.
- Ecosystem services: definition and types.
- Loss of biodiversity.
- Health risks associated with changes in land use and loss of biodiversity.

    - Pandemic risk: emergence and spread of infectious diseases.
    - Food insecurity: desertification, loss of pollinators and reduction of agrobiodiversity.
    - Other risks associated with the loss of biodiversity.
- Mechanisms for the conservation, restoration and sustainable use of nature as health protection mechanisms.
    - Aichi Biodiversity Goals and the 2030 Agenda.
    - Economic instruments.
    - Role of indigenous peoples and local communities.

**Learning outcomes.** At the end of this course students will be able to:

- Assess trends in land use change and biodiversity loss.
- Review the factors driving changes in land use and loss of biodiversity.
- Analyze in an integrated way the impact that changes in land use and the loss of diversity have on human health, from an interdisciplinary perspective.
- Propose actions to protect health based on the critical analysis of policies and strategies for the conservation, restoration and sustainable management of ecosystems.

## MODULE 6. Water Resources and Planetary Health

**Content.** The scarcity of water resources is a growing problem and is mainly due to an increase in human consumption and for agricultural and industrial purposes and, in some areas, less availability due to climate change. The reduction in water quality is explained by increasing contamination by organic compounds, heavy metals and new pollutants such as microplastics. Furthermore, in low-income countries, the lack of infrastructure continues to compromise adequate water sanitation. The scarcity of water resources compromises human health and well-being through different ways: (i) the impact on food security, (ii) the increase in non-infectious diseases related to chemical contamination, (iii) the increase in transmitted infectious diseases by water, and (iv) the unleashing of social tensions and armed conflicts, caused by the control of scarce resources. The objective of this module is to understand global and regional trends in freshwater availability, analyze the causes of growing scarcity and loss of quality, evaluate the effects this has on human health, and evaluate strategies for the sustainable management of freshwater resources to globally guarantee access to clean water worldwide.

- Global evolution of freshwater resources and drivers of scarcity and loss of quality.
- Health effects of scarcity and / or poor quality of fresh water.
    - Food insecurity: limitation of food production.
    - Sanitation and risk of infectious diseases.
    - Chemical pollution: organic compounds, heavy metals and emerging pollutants.
- Access to water and equity.
- Strategies to address water scarcity and growing demand within environmental limits and their relationship to health.
    - Mechanisms for the efficient use of water and new technologies for reuse and purification.
    - Economic instruments for water management.
    - Water resources management.

**Learning outcomes.** At the end of this course students will be able to:

- Analyze global and regional trends in the availability and quality of freshwater resources.
- Argue the various ways in which water scarcity and / or access to low-quality water compromise human health.
- Examine the main causes of the growing shortage of freshwater and the factors that compromise its quality.
- Critically analyze the policies and strategies for the sustainable management of water resources, and evaluate their potential impact on the protection of human health.

## MODULE 7. Global pollution and health

**Contents**. Environmental pollution is responsible for approximately 9 million deaths annually, being the leading environmental cause of morbidity and mortality, and its effects are estimated to be largely underestimated. This burden is very unevenly distributed between countries and populations, disproportionately affecting the population of low- and middle-income countries, where more than 90% of deaths from this cause occur, and vulnerable populations and groups such as indigenous populations, marginal groups, ethnic minorities and children. The sources and routes of exposure to pollution are diverse and include, among others, industry, mining, electricity generation, agriculture, and the combustion of fossil fuels. The objective of this module is to analyze the causes and generating mechanisms of environmental pollution, the effects it has on ecological systems and health, taking into account the differences according to regions and vulnerability groups, and to evaluate the mechanisms for reducing pollution, taking into account cost-benefit, technological and environmental justice factors.

- Global patterns of environmental pollution.
- Environmental pollution treated from ecology, toxicology and environmental epidemiology Sources and routes of exposure to environmental pollution.
- Main risks of environmental pollution for human health.
- Burden of disease associated with environmental pollution.
    - Pollution and social determinants of health.
    - Environmental injustice and highly vulnerable groups.
- Mechanisms of reduction, mitigation and remediation and their relationship with health.
    - Cost-benefit of pollution remediation and reduction policies: limitations and alternatives.
    - Bio-remediation and new technologies.
    - Citizen participation in research and interventions.

**Learning outcomes.** At the end of this course students will be able to:

- Determine the main types of pollution, their sources and routes of exposure for humans.
- Assess the effects of global environmental pollution on health from the point of view of toxicology, environmental epidemiology and ecology.
- Critically compare the uneven distribution of pollution globally and locally and identify populations vulnerable to its effects.
- Critically evaluate pollution reduction mechanisms and policies, considering geographical, social, economic and ethical factors, and their potential impact on global health.

## **MODULE 8. Urbanization and healthy and sustainable cities**

**Contents.** More than half of the world's population currently lives in cities, and predictions indicate that the urban population will continue to grow during the 21st century. This new scenario provides both important challenges and opportunities for humanity. At the health level, poor urban planning can increase the risk of communicable diseases due to inadequate sanitation and overcrowding. Life in cities can increase exposure to unhealthy environments and lifestyle habits, such as higher levels of exposure to air, noise or light pollution, less access to green spaces, insufficient levels of physical activity or less healthy diets, which lead to an increased risk of non-communicable diseases in the urban environment. On the other hand, other important aspects for health, such as access to the health system, tend to be better in urban areas. It is extremely important to plan the urban transition in a manner aligned with the principles of Planetary Health, to avoid possible adverse effects on health of poor urban planning, and to create sustainable and healthy urban centers that, in turn, allow economic development and social of the population. The objective of this module is to analyze the trends in the urbanization process at a global level and the differences by regions, synthesize the challenges and opportunities that this process poses from the perspective of Planetary Health and evaluate the pros and cons of current international initiatives for a sustainable and healthy urbanization.

- Evolution of urbanization patterns.
- Urbanization and impact on the natural environment.
- Urban life and health risks.
    - Urban planning.
    - Lifestyles.
    - Socioeconomic inequalities.
    - Environmental exposures.

- Strategies for planning healthy and sustainable cities.
    - International initiatives for the design of healthy and sustainable cities.
    - Initiatives for healthier and more sustainable urban mobility.
    - Participation and citizen empowerment.
    - Urban environmental justice.

**Learning outcomes.** At the end of this course students will be able to:

- Integrate knowledge about global and regional urbanization processes and apply it to analyze the health effects of the projections envisaged.
- Discuss the challenges to human health and sustainability posed by urbanization processes, both directly and indirectly.
- Contrast the challenges and opportunities that urban life represents for the promotion of health within the framework of Planetary Health.
- Propose different actions to promote healthy and sustainable environments, considering the characteristics of the territory and the populations that inhabit them.
- Critically evaluate initiatives for a sustainable and healthy urbanization, estimating their impact on health, and clearly transmit the main conclusions to specialized and non-specialized audiences.

## MODULE 9. Climate change and health

**Contents.** Climate change is one of the main threats facing humanity in the 21st century. As a result, extreme weather events, such as heat waves, floods, and droughts, are becoming more frequent and intense, leading to human deaths. Likewise, the increase in temperatures can trigger irreversible changes in the functioning of various natural systems essential to guarantee our health. The detrimental health effects of climate change are mediated by multiple pathways including: (i) global warming; (ii) extreme weather events and disasters; (iii) reduction of air quality; (iv) aggravation of allergies; (v) increased risk of infectious diseases; (vi) nutritional deficiencies; (vii) violent conflicts caused by climate-induced migration. The objective of this module is to analyze the evolution of the climate in recent decades and its causes, synthesize the expected effects on the environment and the direct and indirect risks to health, and analyze mitigation and adaptation strategies taking into account economic factors, social and technological and recognizing the different levels of population vulnerability to climate climate depending on local socio-economic and envrionmental characteristics.

- Identification and characterization of climate change.
- Effects of climate change on health.
    - Direct health effects of climate change.
    - Indirect health effects of climate change.
    - Specific effects on vulnerable populations.
- Solutions for mitigation and adaptation to climate change from Planetary Health and challenges for its implementation.
    - Planning of adaptation and resilience measures for health.
    - Mitigation actions and health benefits.
    - Climate justice, poverty and migration.

**Learning outcomes.** At the end of this course students will be able to:

- Assess the causes and possible effects of climate change from a global perspective and analyze the points of no return (“tipping-points”).
- Argue the different complex mechanisms that mediate the health effects of climate change and analyze the sources of climate vulnerability.
- Analyze the health risks, both direct and indirect, of climate change and compare various scenarios based on different carbon emissions and socio-economic pathways
- Critically evaluate adaptation and mitigation strategies to climate change, and analyze how they can contribute to preserving global health.
- Clearly transmit the main conclusions of the analysis of climate change adaptation and mitigation strategies to specialized and non-specialized audiences.

## MODULE 10. Integrative Solutions and Transformative Changes

**Contents.** Planetary Health requires an integrative approach to seek solutions to the complex challenges of the Anthropocene and promote the necessary changes in various sectors of our society to make it sustainable and guarantee our health and that of future generations. To reverse, mitigate and / or adapt and the effects of global environmental change, profound changes are urgently required in our way of consuming and organizing ourselves, as well as technological advances that accelerate mitigation or minimize our impact on ecosystems. The objective of this module is to apply the concept of transformative changes in the framework of Planetary Health, critically evaluate current sustainable development strategies and synthesize transdisciplinary initiatives to promote a healthy and sustainable society.

- Changes needed to ensure planetary health.
- Sustainable development and the 2030 agenda: potentials and limitations, monitoring of progress and failures.
- Science-based policy and research agenda for sustainable development.
- Principles for transformative health change.
    - Ethics, values and equity.
    - Resilience.
    - Measurement of human progress and well-being.
- Mechanisms for integrative transformations in Planetary Health.
    - Governance.
    - Economy and finance: circular economy and degrowth.
    - Individual and collective action.
    - Science and Technology.

**Learning outcomes.** At the end of this course students will be able to:

- Critically analyze the concept of transformative change and contrast it with the concept of incremental change, as well as apply this concept to the different planetary health challenges.
- Establish common key elements in transformative change, such as cultural ethics and values, equality and justice, resilience, and new approaches to measuring well-being.
- Argue the transformative capacity of sustainable development.
- Critically evaluate the 2030 agenda, including analysis of potentials and limitations, updated analysis of progress and barriers to achieving the SDGs.
- Propose strategies to achieve transformative changes in Planetary Health integrating solutions based on the economy and finance, individual and collective action, science and technology and governance.

## MODULE 11. Planetary Health Research: From Hypotheses to Research Protocol

**Contents.** The objective of this module is the planning of the research protocol that will later be developed within the framework of the Master's Final Project. Therefore, its purpose is to familiarize students with the relevant areas for research in Planetary Health and to develop the ability to identify and develop a research protocol in one of these areas. Students will identify and focus on a certain area to address a relevant scientific question and develop a short research protocol. The research area and approach adopted will be consistent with the academic backgrounds of the participants and may range from qualitative approaches to complex quantitative methodologies. Likewise, the inclusion in the project of transversal contributions such as the inclusion of multidisciplinary and transdisciplinary components, the opportunity to capitalize on the SDGs, the recognition of uncertainty and the ability to communicate with those responsible for policies and with society will be promoted.

- Planetary Health Research.
- Preparation of a research protocol in Planetary Health.
    - Formulation of research questions, hypotheses and objectives.
    - Choice of necessary methodologies.
    - Planning data collection and generation of databases.
    - Identification of transversal capacities.
    - Drafting a research protocol.

**Learning outcomes.** At the end of this course students will be able to:

- Define a study question related to one of the main challenges of Planetary Health and formulate relevant research hypotheses.
- Apply the advanced knowledge acquired during the master's degree for planning research in Planetary Health to address the hypotheses formulated, applying criteria of quality, sustainability and social responsibility.
- Propose the most appropriate research methodology to respond to the hypotheses raised and use a diverse set of approaches in the analysis and interpretation of the information to generate new ideas
- Incorporate transversal capacities to said project including multi- and trans-disciplinary
- Assess the utility and viability of the project considering socioeconomic, political and / or cultural factors.

**<u>MODULE 12. Master’s thesis</u>**

**Contents.** The Master’s thesis is the culminating point of the master in Planetary Health. During the completion of the Master’s thesis, the student is expected to integrate and apply the knowledge acquired in the previous modules to develop the research project in the field of Planetary Health defined in the course of module 11. The contents of the Master's thesis are related to research around a real challenge related to Planetary Health, chosen by the student according to their interests and approached in a transdisciplinary way. Commonly, all master's final projects will address the following aspects:

- Implementation of a research project.
- Data collection from primary or secondary sources.
- Evaluation and analysis of the results of the research carried out.
- Presentation of results.
- Writing of scientific articles.
- Publication strategies (professional and informative publications).
- Strategies for oral communication of results.

**Learning outcomes.** At the end of this course students will be able to:

- Develop a research project based on a relevant research question in the field of Planetary Health.
- Apply innovative data collection methodologies that incorporate a multidisciplinary perspective.
- Collect data from primary or secondary sources and analyze them using precise methodologies to respond to the defined objectives.
- Solve dilemmas of recognition and attribution of ideas or works, based on the ethics and integrity of intellectual work.
- Present and defend the results obtained in a format appropriate to the professional field.

**Example of the implementation of the UOC educational model in the MSc: Module 1 ¨Planetary Health: the Response to Anthropocene Challenges**

This modulee has its origins in an optional modulee from the Master of Public Health programme at UPF (2020-21). This modulee has been also adapted for other postgraduate short-courses, including a summer course at the Public Health School in Menorca (September 2021) and a course of the International Summer School in Advanced methods in Global Health (September 2021).

After the first edition of these courses we conducted a co-creation activity during which students were asked to provide feedback on how Planetary Health should be taught in terms of methodology, evaluation, and content as the final activity of the course. The feedback gathered from these past experiences was used to develop the modulee content of the current version of the "Planetary health: the response to the anthropocene challenges"module.

Following the UOC methodology, we structured the module in five challenges, each of them including several activities.

**CHALLENGE 1. Human health and the health of the planet have to go together** (0.5 ECTS)

**Description of the challenge.** Health is a state of complete physical, mental and social well-being and not merely the absence of disease or infirmity. This definition is part of the WHO Constitution and has not been modified since 1948, the year in which it came into force. The climate crisis and the scientific evidence of the Anthropocene have made us see that, with the current economic growth model, the impacts on the Earth's natural systems will make a good part of the societies and human life of many countries unviable, as we know it today. That is why the concept of planetary health has been developed that includes the sustainability of the planet as part of human health. Planetary Health has recently been conceived as the achievement of the highest possible level of health, well-being and equity throughout the world through careful attention to the human systems - political, economic and social - that shape the future of humanity and Earth's natural systems, which define the safe environmental limits in which humanity can thrive. In other words, Planetary Health is the sum of human health and the health of the planet. For this reason, in this challenge we rethink the way we define and understand human health, considering how human activity has impacted the planet and how the climate and environmental crisis influence human health. This concept of planetary health is strongly related to the fact that the responsibilities for the degradation of the planet and its impacts are very unequally distributed, so that the most disadvantaged countries and populations with less responsibility for the climate and biodiversity crisis are the most affected.

**Description of the activities.** This assignment will begin with a first activity in which you are asked to think/analyze what the focus of planetary health is and what its main characteristics are. In order to perform this first activity you will have to view an introductory video about the Anthropocene, an introductory lecture on planetary health, read the executive summary of the Lancet report on planetary health, and the Planetary Health concept of Panel 2 of page 1978; as well as a comment by the Lancet Planetary Health magazine on equity in health and sustainability.

Once you make these views and readings, you will need to write a planetary health definition and identify the main differences compared to the WHO's official health definition (which you need to look for). We also ask you in this activity to try to make the definition of 'planetary health' operational and identify ways to implement the concept (for example, how we can measure planetary health).

Finally, with the baggage acquired, you are proposing a critical understanding of the paradox of improving health and the deterioration of natural systems, in which you will have to explain why there has been a great improvement in human health on a global scale which at the same time coincides with a great deterioration in the health of the planet. For this latter activity, it is requested to answer questions raised in Case 1 study by working on the prominent article 'Safeguarding human health in the Anthropocene epoch'. You can read the optional article 'Untangling the Environmentalist's Paradox: Why Is Human Well-being Increasing as Ecosystem Services Degrade' to finish completing your answers from Case 1 study. As part of this task, you will have to deliver the pound 3 of this CAP in which you are asked to evaluate the case 1 response of one of your fellow subject members, following the heading you will find in this document.

The perspective of the social and economic determinants of human health and planetary health must be present in the different tasks and tasks of challenge 1. In addition to the comment by the Lancet Planetary Health, conceptual models collected in the article by Borrell and Malmusi (2010) and the document. Achieving health: from root causes to fair guesses' by the Commission on Social Determinants of Health will serve as an introduction to understand the approximation of social determinants of health and health inequalities.

Finally, as optional materials of great interest, two recently edited textbooks on planetary health are mentioned which may be of great use throughout the master's degree (Planetary Health: Safeguarding Human Health and the Environment in the Anthropocene; Planetary Health: Protecting Nature to Protect Ourselves.

<u>Activity 1. Writing a definition of the concept of planetary health and identifying the main differences compared to the official WHO health definition.</u>

Estimated dedication time: 2.30 hours

Reading Materials:

- Arlind Boshnjaku. Welcome to the Anthropocene [Internet]. 2012 [citado 23 de febrero de 2022]. Disponible en: https://www.youtube.com/watch?v=fvgG-pxlobk
- Antó, JM. Helsinki Planetary Health Conference in December 2019. [Internet]. 2019. Disponible en: https://www.youtube.com/watch?v=75Tt3PIAlIw.list=PLVpzFDc4BUCRQSh9Gv5hJlRYJPdKO9dF.index=25
- Whitmee S, Haines A, Beyrer C, Boltz F, Capon AG, de Souza Dias BF, et al. Safeguarding human health in the Anthropocene epoch: report of The Rockefeller Foundation–Lancet Commission on planetary health. The Lancet. noviembre de 2015;386(10007):1973-2028.
- Munro A, Boyce T, Marmot M. Sustainable health equity: achieving a net-zero UK. Lancet Planet Health. diciembre de 2020;4(12):e551-3. 4. TEDx Talks. TEDxCanberra - Will Steffen - The Anthropocene [Internet]. 2010 [citado 23 de febrero de 2022]. Disponible en: https://www.youtube.com/watch?v=ABZjlfhN0EQ

<u>Activity 2. Case study to understand the paradox of improving health and deteriorating natural systems</u>

Estimated dedication time: 3 hours

Reading Materials:

- Whitmee S, Haines A, Beyrer C, Boltz F, Capon AG, de Souza Dias BF, et al. Safeguarding human health in the Anthropocene epoch: report of The Rockefeller Foundation–Lancet Commission on planetary health. The Lancet. noviembre de 2015;386(10007):1973-2028.

<u>Activity 3. Peer-evaluation. Evaluate case study responses (from activity 2) by another student</u>

Estimated dedication time: 1.30 hours

Reading materials: N/A

**CHALLENGE 2. How has human activity altered the planet's natural systems? (**1.5 ECTS)

**Description of the challenge.** According to the scientific community, we are now in a new geological epoch called the Anthropocene that you are beginning to recognize, but the changes that make up the Anthropocene are multiple and complex and you will need to develop a more detailed analytical understanding. According to geologists and other earth scientists, human activity is leaving a pervasive and persistent imprint on the Earth. This has prompted an intense debate about whether these changes deserve recognition as a new unit of Geologic Time known as the Anthropocene. Anthropogenic markers of

functional changes in the planetary system identified through stratigraphic records include: manufactured materials in sediments; global peaks in radionuclides and radioactive fallout particles; alteration of the carbon, nitrogen and phosphorus cycles; human disruption of the climate system with global warming; and biotic changes including the massive extinction of species among others. These combined signals make the Anthropocene stratigraphically different from the Holocene and earlier epochs. The impacts of the activity on the planet's systems that characterize the Anthropocene are of paramount importance for human health to the point that it has led us to the concept of planetary health. The understanding of the Anthropocene is essential to understand some of the great challenges that humanity faces such as the climate crisis.

**Description of the activities.** In this second challenge you will become familiar with the concepts of Anthropocene and Borders Planetary through vision of two recordings. We'll ask you to search first Quick on the Anthropocene concept and share it with your colleagues in the 'debate' section in the classroom. After viewing videos, you'll have to make one description of the concept and characteristics of the Anthropocene using theoretical information and empirical. Once this concept has been introduced, we will go into its origins and evolution with particular attention to its scientific basis. That's why you need to read partially or in a way complete some leading articles in this field proposed in resources Learning.

You will then become familiar with how $CO_2$ and gas emissions are measured with effect de And you'll find an App that allows you to measure your $CO_2$ emissions and compare with the average per capita emissions in your country. Next, once the concept has been introduced from the Anthropocene, you will work and answer the questions raised in Case 2 study on The impact of health systems on the planet's health.

In this challenge, we will look at how scientific and humanistic knowledge comes from multiple disciplines converge when showing which current answers should be for maintain a sustainable planet and a resilient society that allows prosperity present and future generations.

Finally, through the 'debate' section of the classroom, you will share your perspective on the role of health system and professionals faced with the challenges of the Anthropocene.

Activity 1. Participate in the debate in the virtual campus and propose a definition about the Anthropocene's concept.

Estimated time: 1 hour

Reading materials:

- TEDx Talks. TEDxCanberra - Will Steffen - The Anthropocene [Internet]. 2010 [citado 23 de febrero de 2022]. Disponible en: https://www.youtube.com/watch?v=ABZjlfhN0EQ
- World Economic Forum. Beyond the Anthropocene | Johan Rockström [Internet]. 2017 [citado 23 de febrero de 2022]. Disponible en: https://www.youtube.com/watch?v=V9ETiSaxyfk

Activity 2: Develop the concept and characteristics of the Anthropocene using the theoretical and empirical information provided in the reading material of this one challenge and the previous one. Include a diagram.

Estimated time: 6 hours

Reading material:

- Folke C, Polasky S, Rockström J, Galaz V, Westley F, Lamont M, et al. Our future in the Anthropocene biosphere. Ambio. abril de 2021;50(4):834-69.
- Waters CN, Zalasiewicz J, Summerhayes C, Barnosky AD, Poirier C, Gałuszka A, et al. The Anthropocene is functionally and stratigraphically distinct from the Holocene. Science. 8 de enero de 2016;351(6269):aad2622.
- Steffen W, Richardson K, Rockström J, Cornell SE, Fetzer I, Bennett EM, et al. Sustainability. Planetary boundaries: guiding human development on a changing planet. Science. 13 de febrero de 2015;347(6223):1259855.

- Steffen W. Introducing the Anthropocene: The human epoch: This article belongs to Ambio's 50th Anniversary Collection. Theme: Anthropocene. Ambio. octubre de 2021;50(10):1784-7.
- Davison N. The Anthropocene epoch: have we entered a new phase of planetary history? The Guardian [Internet]. 30 de mayo de 2019 [citado 23 de febrero de 2022]; Disponible en: https://www.theguardian.com/environment/2019/may/30/anthropocene-epoch-have-we-entered-a-new-phase-of-planetary-history

Activity 3: Find an App that allows you to measure your $CO_2$ footprint. Describe your emissions and compare them to the average per capita emissions from the country where you live.

Estimated time: 4 hours

Reading material:

- Measuring Carbon Footprints | Mathematics of Planet Earth [Internet]. [citado 23 de febrero de 2022]. Disponible en: http://mpe.dimacs.rutgers.edu/2013/05/21/measuring-carbon-footprints/
- Ritchie H, Roser M. $CO_2$ and Greenhouse Gas Emissions. Our World in Data [Internet]. 11 de mayo de 2020 [citado 23 de febrero de 2022]; Disponible en: https://ourworldindata.org/greenhouse-gas-emissions

Activity 4: Case study about the environmental impacts of health systems

Estimated time: 4 hours

Reading material:

- Malik A, Lenzen M, McAlister S, McGain F. The carbon footprint of Australian health care. Lancet Planet Health. enero de 2018;2(1):e27-35.

Activity 5: Sahre in the debate section of the virtual campus, your perspective on the role of the health system and professionals in front of the challenges of the Anthropocene.

Estimated time: 2 hours

Reading material: N/A

**CHALLENGE 3. What proportion of global human health loss would you say is attributable to environmental factors? (**1.2 ECTS)

**Description of the challnge.** Pollution is the leading environmental cause of disease and premature death in the world today. Diseases caused by pollution were responsible for approximately 9 million premature deaths in 2015 – representing 16% of deaths worldwide – three times more deaths than from AIDS, tuberculosis, and malaria combined and 15 times more than from all of them. wars and other forms of violence. In the most affected countries, pollution-related illnesses are responsible for more than one death in four. Pollution disproportionately affects the most socioeconomically disadvantaged and also the most vulnerable. Nearly 92% of pollution-related deaths occur in low- and middle-income countries, and in all countries, illnesses caused by pollution are more prevalent among minorities and marginalized populations. Children are at high risk of pollution-related illnesses, even when exposures to pollutants occur at extremely low doses during windows of vulnerability to the womb during pregnancy and in early childhood, which can affect health and even result in death. in death, disease and disability during childhood and the rest of life.

**Description of the activities.** The WHO has estimated that about 25% of disability-adjusted life years (DALYs) are attributable to environmental factors. To begin this challenge, you will view the presentation by Dr. Maria Neira, director of public health at the WHO, where she describes what environmental health is and exposes the factors that compromise it, highlighting the importance of dealing with its impacts. From

here you will develop a definition of environmental (human) health and identify the main environmental factors that influence human health. Knowing the environmental risk factors and their impacts will allow you to develop strategies and interventions that allow coping through health promotion and prevention.

Studies to measure the impacts attributable to exposures to risk factors such as the Global Burden of Disease (GBD) study have allowed the Lancet Commission on Pollution and Health to describe the wide scope of these impacts and place on the radar those potentially very important exposures, but still insufficiently known (pollutome). These two platforms (GBD and Lancet Commission) work in parallel on the subject "Interdisciplinary Approaches for Planetary Health". Using the data provided by the GBD you will have to select a high-income country and a low-income country and compare the impact of environmental factors on human health. For this activity, see section 1 ("The burden of disease attributable to environmental factors") of Landrigan et al. The Lancet Commission on Pollution and Health, with special attention to Table 1 and the graphic-concept of the "pollutome".

Continuing with the report of the Landrigan et al. The Lancet Commission on Pollution and Health on Pollutome, you will have to critically analyze the effects of pollution on the brain with special attention to the effects in the first stages of life. Read Panel 4 and the section "developmental neurotoxicant". Next, you will have to analyze and interpret the solutions and interventions to control and prevent the effects of pollution on health, reviewing Panel 3 (WHO's program on pollution and health" and section 4 "effective interventions against pollution" by Landrigan et al. The Lancet Commission on Pollution and Health.

Finally, in groups (of about 4-5 people who will form freely) you will choose a city that you know, have lived in, or are familiar with and you will propose 5 actions that reduce the effects of pollution on human health, considering social inequalities and the most vulnerable groups. For this exercise, you can consider the work of Mujer N et al. on environmental impacts on human health in Barcelona; or look for similar studies, if they exist, on the City they have chosen. In addition to submitting a document to be evaluated, the groups will share a brief summary of their results/conclusions in the classroom debate.

<u>Activity 1: Develop a definition on environmental (human) health and identify which are the main environmental factors that influence human health.</u>

Estimated time: 4 hours

Reading material:

- ISEE Global Education Channel. MARIA NEIRA - WHO Global Strategy on Health, Environment and Climate Change [Internet]. 2018 [citado 23 de febrero de 2022]. Disponible en: https://www.youtube.com/watch?v=FTBgecH8T-M
- Landrigan PJ, Fuller R, Acosta NJR, Adeyi O, Arnold R, Basu NN, et al. The Lancet Commission on pollution and health. Lancet. 3 de febrero de 2018;391(10119):462-512.

<u>Activity 2: Using the data provided by GBD select one high-income country and low-income country and compare the impact environmental factors to human health.</u>

Estimated time: 4 hours

Reading material:

- Global Burden of Disease (GBD 2019) [Internet]. Institute for Health Metrics and Evaluation. 2014 [citado 23 de febrero de 2022]. Disponible en: https://www.healthdata.org/gbd/2019
- Landrigan PJ, Fuller R, Acosta NJR, Adeyi O, Arnold R, Basu NN, et al. The Lancet Commission on pollution and health. Lancet. 3 de febrero de 2018;391(10119):462-512.

<u>Activity 3: Reflect on the concept of Pollutome, that you can find at the Lancet Commission on Pollution and Health report, and searches for examples about the areas of uncertainty it describes.</u>

Estimated time: 2 hours

Reading material:

- Landrigan PJ, Fuller R, Acosta NJR, Adeyi O, Arnold R, Basu NN, et al. The Lancet Commission on pollution and health. Lancet. 3 de febrero de 2018;391(10119):462-512.

<u>Activity 4: Analyze and critically interpret solutions and interventions to control and prevent the effects of pollution on human health.</u>

Estimated time: 2 hours

Reading material:

- Landrigan PJ, Fuller R, Acosta NJR, Adeyi O, Arnold R, Basu NN, et al. The Lancet Commission on pollution and health. Lancet. 3 de febrero de 2018;391(10119):462-512.

<u>Activity 5: In assigned groups choose a city you know or the one that you live and suggest 5 actions that decrease the effects of pollution in human health by considering social inequalities and the most vulnerable groups.</u>

Estimated time: 6 hours

Reading materials:

- Whitmee S, Haines A, Beyrer C, Boltz F, Capon AG, de Souza Dias BF, et al. Safeguarding human health in the Anthropocene epoch: report of The Rockefeller Foundation–Lancet Commission on planetary health. The Lancet. noviembre de 2015;386(10007):1973-2028
- Mueller N, Rojas-Rueda D, Basagaña X, Cirach M, Cole-Hunter T, Dadvand P, et al. Urban and Transport Planning Related Exposures and Mortality: A Health Impact Assessment for Cities. Environmental Health Perspectives. enero de 2017;125(1):89-96.

**CHALLENGE 4. The problem is not the environment. The problem is our socioeconomic models that spoil human health and that of the planet.** (0.6 ECTS)

**Description of the challenge.** Current trends of degradation of the planet's natural systems have the potential to reverse the gains in health achieved so far and destabilize human civilization. That is why a new approach to health problems is needed. It is no longer just about understanding environmental factors as a major cause of health loss, as we have seen in the previous challenge, but about understanding the bidirectional relationship between human health and the health of the planet. This change in focus has two important consequences. On the one hand, it allows us to broaden the range and understanding of the impacts of the environment on human health, such as how climate change impacts human health. On the other hand, it allows us to understand and respond to the fact that our actions, both those related to health and those related to other sectors, have an impact on the health of the planet and feedback on human health. Thus, the planetary health approach includes an agenda of multisectoral policies and actions to restore natural resources, preserving and improving human health. The starting point is the understanding of problems based on complexity, multidisciplinary and urgency so that we can face new challenges such as conceptual ones (see challenge 1), those of knowledge (challenges 2,3, 4) and those of implementation (challenge 5). Fortunately, we have enough scientific evidence to urgently establish far-reaching transformative measures to help stop global warming and biodiversity loss while improving human health.

**Description of the activities.** After seeing, in challenge 3, the problems of environmental health as we have considered them in recent decades (the unfinished agenda of environmental health), we now focus on a vision of human health from the perspective of the Anthropocene. The approach to this challenge begins by identifying the main health problems due to the impacts of changes in natural systems typical of the Anthropocene. These problems are analyzed in detail in the report of the Lancet Commission on Planetary

Health (Safeguarding human Health in the Anthropocene). They are also described in Part 2 (Chapters 5-10) of the book by Mayers and Frumkin (Planetary Health). With this knowledge, you will have to identify and describe the main health problems due to the impacts of changes in natural systems typical of the Anthropocene. Based on this knowledge, you will focus on the city selected in deliverable 2 of challenge 3, and individually, you will briefly analyze the importance of health problems due to the impacts of changes in the Earth's natural systems.

One of the key areas of work is the climate crisis and its orientation towards solutions, so continuing with the selected city you will have to propose 4 actions or strategies aimed at responding to global warming, including adaptation and mitigation actions. The health impacts of global warming are of special importance to the planetary health agenda. To better understand how climate change impacts our health, you will watch the Climate Change is Affecting Our Health video. Is There a Cure? by Jonathan Patz. To get a systematic view of the extent of these impacts, you will use the 2020 Lancet Countdown on Health and Climate Change report (section I: impacts). In relation to the solutions you will read the sections “Charting a course for the future” pages 1997-2007, of the Lancet Commission on Planetary Health (Safeguarding human Health in the Anthropocene). Chapter 10 of Mayers and Frumkin's book is devoted to the health effects of climate change.

Chapter 10 of the book by Mayers and Frumkin (Planetary Health) and optional materials (Report IPCC and WHO for COP26 will help you to finish understanding the impact of global warming and climate change over global health. They'll serve you to think strategies and/or actions to deal with the planetary crisis situation we are experiencing. Chapter 10 of book by Mayers and Frumkin is dedicated to the health effects of climate change.

<u>Activity 1: Describe the major health problems due to the changes in natural systems as part of the Anthropocene epoch.</u>

Estimated time: 2.30 hours

Reading materials:

- Whitmee S, Haines A, Beyrer C, Boltz F, Capon AG, de Souza Dias BF, et al. Safeguarding human health in the Anthropocene epoch: report of The Rockefeller Foundation–Lancet Commission on planetary health. The Lancet. noviembre de 2015;386(10007):1973-2028
- Myers SS, Frumkin H. Planetary Health - Protecting Nature to Protect Ourselves [Internet]. Island Press. 2020 [citado 24 de febrero de 2022]. Disponible en: https://islandpress.org/books/planetary-health

<u>Activity 2: Briefly analyze the importance of health problems due to the impacts of changes in the natural systems in the Anthropocene, in the city you chose in challenge 3.</u>

Estimated time: 2.30 hours

Reading material:

- Masson-Delmotte VP, Zhai HO, Pörtner D, Roberts J. Summary for Policymakers. In: Global Warming of 1.5°C. [Internet]. 2018 [citado 24 de febrero de 2022]. Disponible en: https://www.ipcc.ch/sr15/
- COP26 Special Report on Climate Change and Health [Internet]. [citado 23 de febrero de 2022]. Disponible en: https://www.who.int/publications-detail-redirect/cop26-special-report

<u>Activity 3: Continuing with the city you selected, suggest four types of actions or strategies aimed at responding to global warming by analysing their expected effects on human health.</u>

Estimated time: 3 hours

Reading material:

- TEDx Talks. Climate Change is Affecting Our Health. Is There a Cure? | Jonathan Patz | TEDxOshkosh [Internet]. 2018 [citado 23 de febrero de 2022]. Disponible en: https://www.youtube.com/watch?v=r_Dpkn_wi54
- Watts N, Amann M, Arnell N, Ayeb-Karlsson S, Beagley J, Belesova K, et al. The 2020 report of The Lancet Countdown on health and climate change: responding to converging crises. The Lancet. 9 de enero de 2021;397(10269):129-70.
- Whitmee S, Haines A, Beyrer C, Boltz F, Capon AG, de Souza Dias BF, et al. Safeguarding human health in the Anthropocene epoch: report of The Rockefeller Foundation–Lancet Commission on planetary health. The Lancet. noviembre de 2015;386(10007):1973-2028


**CHALLENGE 5. We need urgent social transformations to respond to the challenges of the Anthropocene. The future is now (**Credits: 1.2 ECTS)

**Description of the challenge.** As the sixth UN State of the World Environment Report (GEO-6) has shown, our species is at a crossroads. We can launch into a new era of sustainable development, as proposed by the UN SDG 2030 Agenda, or continue with current trends of huge environmental and social upheavals, such as the climate crisis. The UN report The Future is Now identifies six essential entry points to accelerate the necessary transformation: i) Well-being and human capacities, ii) Sustainable and fair economies, iii) Food systems and nutrition patterns, iv) Energy decarbonization and access universal, v) urban and peri-urban development and vi) environmental common goods. The report also identifies four "levers" that can be applied to these critical entry points to strike a balance between human well-being and its social and environmental costs: i) Governance, ii) Economics and finance, iii) Individual and collective action, and finally iv) Science and technology. Applying this approach to health challenges makes it possible to develop responses to planetary health challenges. As GEO-6 Co-Chairs Joyeeta Gupta and Paul Eakins have highlighted: A healthy people, a healthy planet, and a healthy economy can be mutually supportive, and our current scientific knowledge justifies political action. Continuing with the current models of environmental policy is necessary, but it is often completely insufficient to address current systemic ecological problems. We urgently need new transformative and holistic solutions to contribute to a healthy future.

**Description of the activities.** The current climate crisis and other natural systems need effective and urgent solutions. In this challenge we start from the main health problems due to the impacts of changes in natural systems typical of the Anthropocene, and some of the possible solutions, which we have seen in previous challenges, to focus on our own intervention policies and strategies of the planetary health approach.

Now we want you to become familiar with the science-based understanding of possible solutions and to be inspired by those who advocate and apply these solutions. Therefore, starting from the main health problems due to the impacts of changes in natural systems typical of the Anthropocene, summarized in challenge 4, you will have to identify and briefly describe some policies and intervention strategies typical of the planetary health approach. (activity 1).

With this background, we address the UN report "The Future is Now", which was prepared as a reference report to advise those involved in the governance of the Sustainable Development Goals (SDGs), developing the contribution of scientific knowledge in the development of transformative change proposals. Using the report "The Future is Now", you will have to describe and critically comment on the model of transformations proposed in this report that interrelates the "levers" of change with the systems that need to be transformed (activity 2). Therefore, you will have to view the videos "Sustainable Development Goals: Improving Life Around the World" and the video: "The Future is Now: Science for Achieving Sustainable Development".

Next, with the knowledge acquired, you will address a case about the transformation of food systems.

Finally, you will return to deliverable 2 of challenge 3 and select one of the proposed actions and develop it by making suggestions that respond to the three types of challenges described in the Lancet report (conceptual, information and governance).

Activity 1: Identify and briefly describes some of the intervention policies and strategies from the planetary health approach.

Estimated time: 3.30 hours

Reading material:

- Whitmee S, Haines A, Beyrer C, Boltz F, Capon AG, de Souza Dias BF, et al. Safeguarding human health in the Anthropocene epoch: report of The Rockefeller Foundation–Lancet Commission on planetary health. The Lancet. noviembre de 2015;386(10007):1973-2028
- World Economic Forum. Beyond the Anthropocene | Johan Rockström [Internet]. 2017 [citado 23 de febrero de 2022]. Disponible en: https://www.youtube.com/watch?v=V9ETiSaxyfk
- TEDx Talks. Why it's time for «Doughnut Economics» | Kate Raworth | TEDxAthens [Internet]. 2014 [citado 23 de febrero de 2022]. Disponible en: https://www.youtube.com/watch?v=1BHOflzxPjI

Activity 2: Critically describe and comment on the transform model proposed in 'The Future is Now' report.

Estimated time: 3.30 hours

Reading material:

- Independent group of Scientist. Global Sustainable Development Report 2019: The Future is Now – Science for Achieving Sustainable Development [Internet]. 2019. Disponible en: https://sdgs.un.org/es/node/24576
- UN DESA. The Future is Now: Science for Achieving Sustainable Development [Internet]. 2019 [citado 23 de febrero de 2022]. Disponible en: https://www.youtube.com/watch?v=WllsvUG3uzQ

Activity 3: Case study about the food systems with a focus on planetary health

Estimated time: 4 hours

Reading material:

- Today's Solutions for the Future of Food. En: Planetary Health Case Studies: An Anthology of Solutions [Internet]. Island Press; 2020 [citado 23 de febrero de 2022]. Disponible en: https://drive.google.com/file/d/1YWNsc1rBJxUFnxx7x12w6LguN4zD568q/view

Activity 4: Select one of the actions you have proposed in challenge 3 and develop it by making suggestions that respond to the three types of challenges (conceptuals, information and governance).

Estimated time: 2 hours

Reading material:

- Whitmee S, Haines A, Beyrer C, Boltz F, Capon AG, de Souza Dias BF, et al. Safeguarding human health in the Anthropocene epoch: report of The Rockefeller Foundation–Lancet Commission on planetary health. The Lancet. noviembre de 2015;386(10007):1973-2028
